# Identification of Griffon Vulture’s Flight Types Using High-Resolution Tracking Data

**DOI:** 10.1007/s41742-018-0093-z

**Published:** 2018-05-14

**Authors:** Sam Khosravifard, Valentijn Venus, Andrew K. Skidmore, Willem Bouten, Antonio R. Muñoz, Albertus G. Toxopeus

**Affiliations:** 1 Faculty of Geo-Information Science and Earth Observation (IT1C), University of Twente, PO Box 217, 7500 AE Enschede, The Netherlands; 2 Computational Geo-Ecology, Institute for Biodiversity and Ecosystem Dynamics (IBED), University of Amsterdam, PO Box 94248, 1090 GE Amsterdam, The Netherlands; 3 Biogeography, Diversity and Conservation Research Team, Department of Animal Biology, Faculty of Sciences, University of Malaga, 29071 Malaga, Spain

**Keywords:** Animal movement, Animal tracking, Collision, Gliding, Linear soaring, Slope soaring, Spain, Spiral gliding, Telemetry, Wind turbine

## Abstract

Being one of the most frequently killed raptors by collision with wind turbines, little is known about the Griffon vulture’s flight strategies and behaviour in a fine scale. In this study, we used high-resolution tracking data to differentiate between the most frequently observed flight types of the Griffon, and evaluated the performance of our proposed approach by an independent observation during a period of 4 weeks of fieldwork. Five passive flight types including three types of soaring and two types of gliding were discriminated using the patterns of measured GPS locations. Of all flight patterns, gliding was classified precisely (precision = 88%), followed by linear and thermal soaring with precision of 83 and 75%, respectively. The overall accuracy of our classification was 70%. Our study contributes a baseline technique using high-resolution tracking data for the classification of flight types, and is one step forward towards the collision management of this species.

## Background

Flight and foraging behaviour, and migration of the Griffon vulture (*Gyps fulvus,* Hablizl, 1783) have been well studied (Bildstein et al. 2009; Duriez et al. 2014, García-Ripollés et al. 2011; Houston 1974) (see “Appendix”). However, little is known about the fine-scale flight and motion capacity of this species, which is on the top list of most frequently killed raptors by collision with wind turbines in southern Spain (Barrios and Rodríguez 2004).

Flight type plays an important role in collision risk with wind turbines, especially when associated with hunting and foraging strategies of big raptors (Marques et al. 2014). Hoover and Morrison (2015) highlighted that soaring flight, which needs strong wind and occurs in rotor-swept zone of wind farm, is a factor explaining the high collision rate of raptors.

The motion capacity of an individual is its ability to move in various ways or modes either by its own locomotion or by externally vectored via physical means (e.g., winds, water flow, etc.) or by other organisms (e.g., wingless flower mites traveling on foraging bees) (Holyoak et al. 2008). Generally, a movement paradigm was introduced as the interplay amongst the four basic mechanistic components: external factors affecting movement, internal state (i.e., why move?), navigation capacity (i.e., where and when to move?) and motion capacity (i.e., how to move?) (Holyoak et al. 2008; Nathan et al. 2008). A more detailed understanding of the motion capacity of flying birds has been developed in many ornithological studies (Cone 1962; Dhawan 1991; Pennycuick 1971, 1972; Tucker 2000; Videler 2005). Soaring and gliding are the two most common types of flight among raptors and have been at the centre of many studies since the first attempt to understand raptors’ flight behaviour in 1913 (Dhawan 1991). However, a major challenge underlying studies of movement type is of a methodological nature, related to data collection and the methods used to classify the movement patterns.

With respect to data collection, researchers have traditionally used direct observation as a method to monitor birds, as well as to elucidate and describe flight phenomena (Pennycuick and Scholey 1984). Bildstein et al. (2009), for example, used this method during the autumns of 2004–2007 to determine Griffon vultures’ flight types during migration. Not losing sight of a species is the most challenging part of this traditional type of research (Pennycuick 1973), but this has now been solved by telemetry techniques. These methods provide practical insight into wildlife movements (for instance see Harel et al. 2010; Bouten et al. 2013; López-López et al. 2013).

Techniques for studying free-living birds’ behaviour have advanced and flourished since these earlier attempts (Roy and Hart 1963). Since then, technologies including radar (Konrad et al. 1968), radio (Schemnitz et al. 1969), satellite and Global Positioning System (GPS) tracking (Biro et al. 2002; Nowak et al. 1990; Weimerskirch et al. 2002) have been deployed. Recent advances in telemetry techniques, such as extensive use of bio-loggers with GPS, have enabled spatiotemporal data to be collected on vertebrates with ever-increasing accuracy as well as density of data points (Tomkiewicz et al. 2010).

Much research has been conducted via the classification of movement patterns to solve the difficulties of dealing with large datasets and their interpretation (e.g., Güting et al. 2010). These methods, however, have been used mainly to analyse movement in two dimensions (i.e., *x* and *y*) (Giannotti et al. 2008; Güting and Schneider 2005; Long and Nelson 2013) and mostly at coarse temporal resolution (i.e., daily or hourly movements) to determine home range, dispersal and migration routes (Calenge et al. 2009; Kranstauber et al. 2012; López-López et al. 2013; Mandel et al. 2008; Smouse et al. 2010).

Research to date indicates that the Griffon vulture exhibits mainly passive flight types (i.e., various kinds of soaring and gliding) using air currents, as well as occasional flapping when necessary (Bildstein et al. 2009; Dhawan 1991). Moreover, using accelerometer data, Halsey et al. (2009) proved that the species rarely flaps except during take-off or landings in non-migratory movement. Since soaring birds such as the Griffon vulture are not capable of maintaining constant altitude by flapping flight alone (Newton 2010; Shepard et al. 2011) and it has also been shown by Bildstein et al. (2009) that the flapping rate in the Griffon vulture is very low (i.e., mean of 1.2 flaps per 30 s), we made a basic assumption in this study that the flapping rate during daily flights can be considered negligible in non-migratory movement.

Our study utilised collection methods using GPS-logger technology. Based on the high-resolution tracking data only, we developed and tested a baseline method to differentiate passive flight in three spatial dimensions (i.e., *x*, *y* and *z*) to classify these flight types of the Griffon vulture. This study is one step forward to have more insight into flight behaviour which may play a role in collision risk.

## Materials and Methods

### Study Area and Species

**Fig. 1:**
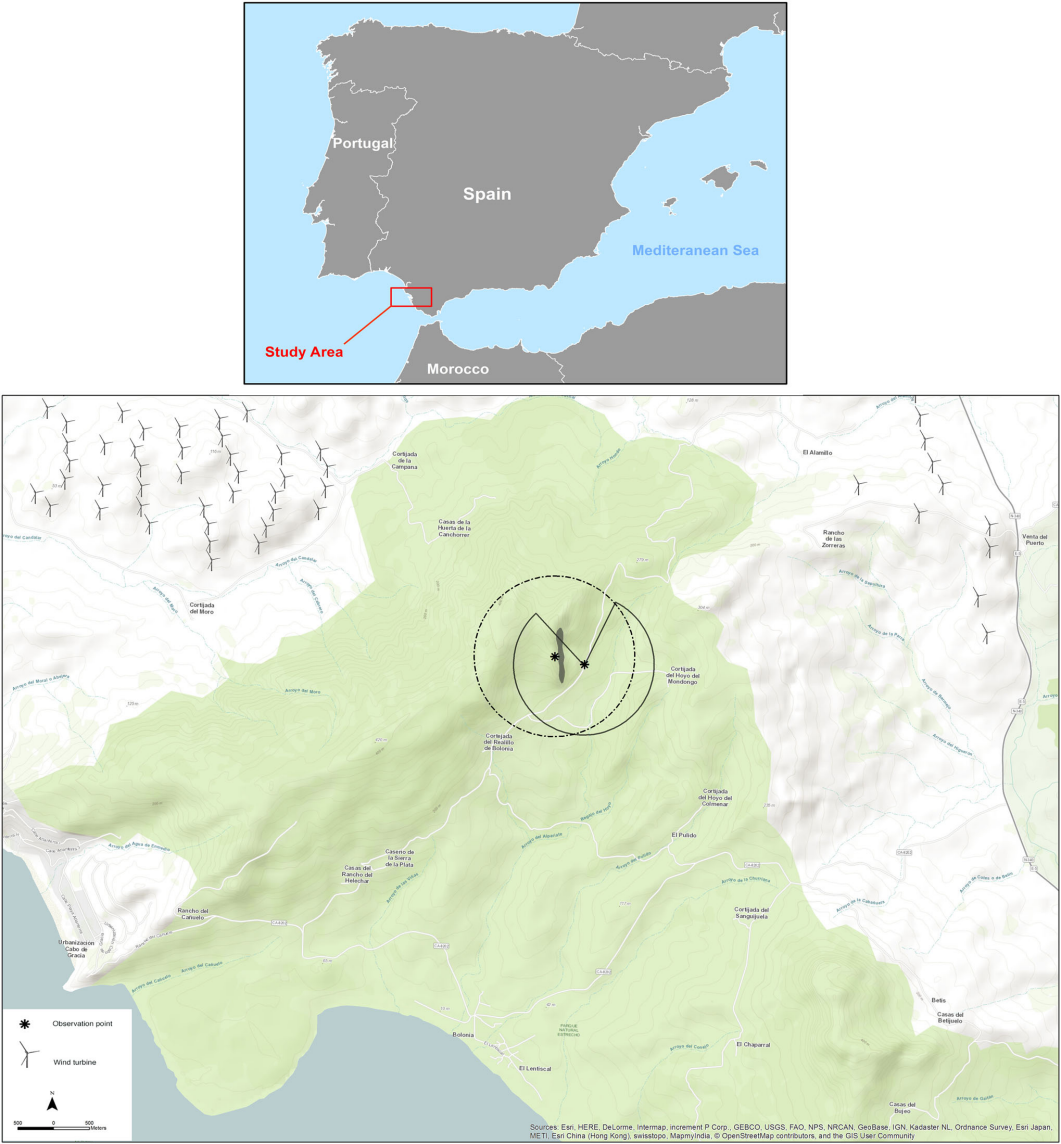
The study area in province of Cádiz, south Spain: the grey polygon (bottom) is the location of colony site and the asterisk symbols show the location of observers. The observers’ angle of view is shown in solid and dashed line

Our study area in southern Spain is part of the natural park El Estrecho, in Tarifa, Andalucía region, and is located on the northern side of the Strait of Gibraltar (36°07′–36°06′N, 5°45′–5°46′W). The Strait of Gibraltar is the shortest sea crossing between Europe and Africa and is a well-known migratory bottleneck for soaring birds (Zalles and Bildstein 2000). In this area, Ferrer et al. (2012) reported the highest collision rates ever published for birds (1.33 deaths/turbine/year) with the Griffon vulture being the most frequently killed species (0.41 deaths/turbine/year). An escarpment with north–south direction, 4 km away from the Strait of Gibraltar, is a location of Griffon vulture’s colony, consisting of approximately 65 breeding pairs (Del Moral 2009) The population is surrounded by several other breeding colonies, consisting of approximately 320 pairs so the area is persistently used by vulture during their local movements (De Lucas et al. 2012) and is encompassed by 25 wind farms, consisting of 491 operating turbines. Figure 1 shows the study area, location of wind turbines and the colony.

A Griffon vulture was captured using a foot snare. The bio-logger was attached to it as a backpack using a harness made of Teflon ribbons with one strap fitting across each wing and another strap below the crop (Kenward 2000). The capture and release took place on September 11, 2010. In addition, distinctive yellow patagial markers, with unique combination of numbers and letters (i.e., 9FJ) were attached to both wings. This method was proved to be harmless to the bird with no changes in its normal behaviour (Reading et al. 2014). The captured Griffon vulture was a male, sub-adult, and with a body mass of about 7 kg.

Collision risk may also be influenced by behaviour associated with a specific sex or age. Although it is reported that young vultures were not especially vulnerable to collisions compared with the other age classes (Barrios and Rodríguez 2004; Marques et al. 2014), de Lucas et al. (2012) demonstrated that among 117 killed vultures by collision with turbines, 74.36% (87) were juveniles and 25.64% (30) were matures and adults. Additionally, to the best of our knowledge, no information has been published about correlation between sex and collision rate of the Griffon vulture.

### Tracking Device

We used the Bird Tracking System developed at the University of Amsterdam (Bouten et al. 2013). The key features of its bio-logger are rechargeable solar batteries, low weight style (45 g, < 0.6% of a Griffon vulture’s body mass), two way data-communication, four megabytes flash memory (capable of sorting 60,000 GPS fixes) and the GPS tag with high resolution temporal intervals from 3 s up to 7200 s (see http://www.uva-bits.nl for more information). In this study, we used GPS fixes and their properties to differentiate between the flight types.

### Collecting Data from a Free-Ranging Vulture

Tracking data were retrieved for 27 days between May and July 2013. This period was a part of breeding season of the bird. During this time, we also undertook fieldwork observations independent of the tracking dataset. We used a camera recorder synchronized to Universal Time Coordinated (UTC) time with Garmine eTrex Summit GPS along with direct visual observations to note the times and flight types simultaneously. The observations were made by two observers during daylight hours with the aid of 10 × 42 binoculars and a 20–60× telescope spot. We conducted a filed survey to select the observation locations with a wide angle of view in almost centre of the escarpment: one up and the other down on the cliff with almost 360° and 270° angle of view, respectively. To motivate the Griffon vulture to fly, carrion was dumped on the ground. Additionally, observation points were selected to provide a wide field of view of the tagged bird with the yellow patagial markers on the dorsal and ventral surfaces of wings.

### Data Preparation

Although we had set the measurement interval of the GPS tracker to 3s, the retrieved datasets consisted of various intervals. Therefore, to prepare the final dataset, we extracted 11 days of collection data with a three-second interval, yielding 66,766 data points. The instrument recorded several properties for each point including time, geographic coordinates, altitude, and instantaneous velocity in three directions (*x*, *y* and *z*). Based on this raw data, we calculated the distance, cumulative distance, average altitude, altitude difference and direction of motion between all successive GPS fixes. To discriminate between flying and non-flying modes, we considered speed of movement and calculated the first non-static points with a speed > 4 m/s (Nathan et al. 2012).

### Flight Types

**Fig. 2:**
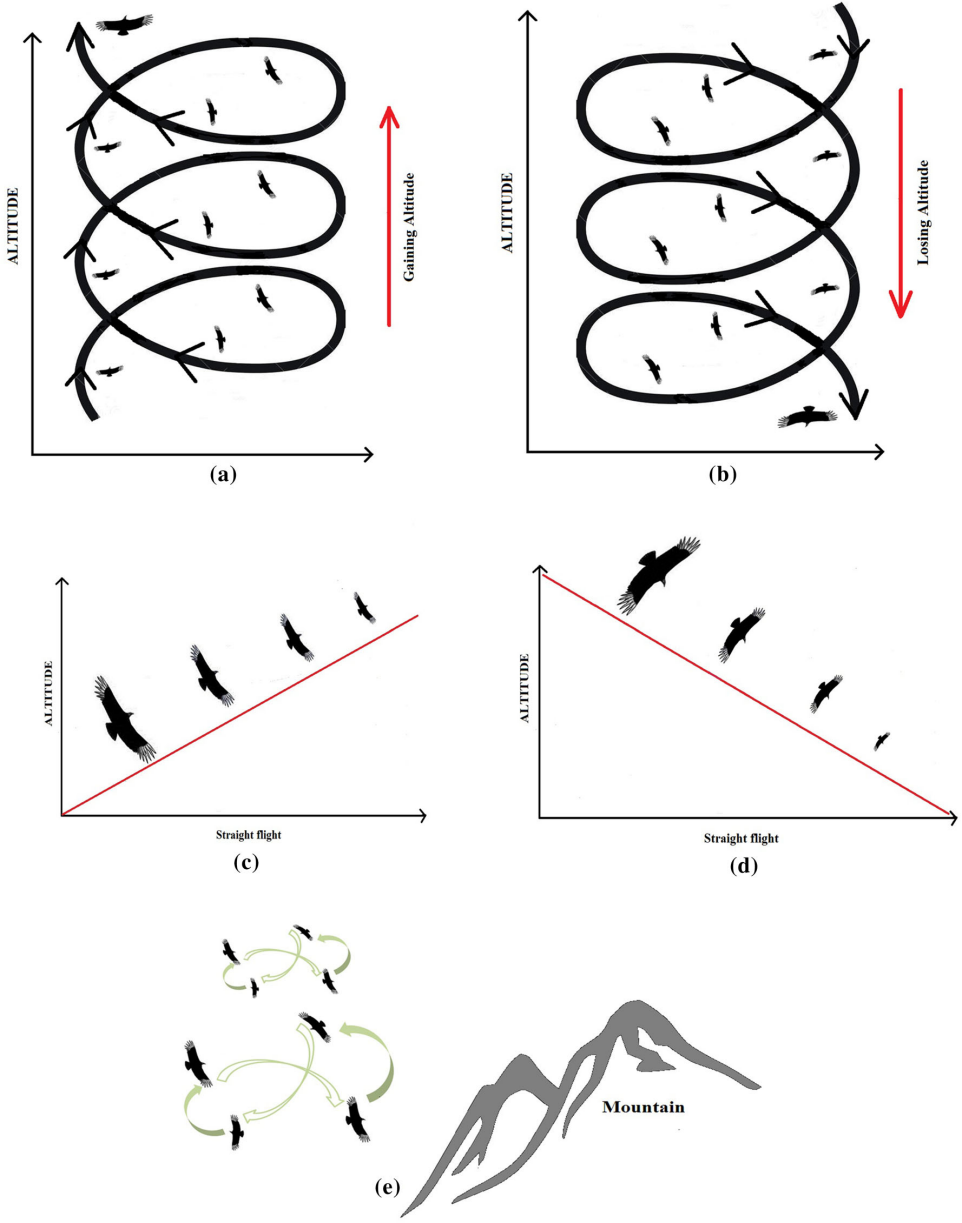
Thematic illustration of the Griffon vulture’s different flight patterns **a** thermal soaring, **b** spiral gliding, **c** linear soaring, **d** gliding, **e** slope soaring

This paper focuses on five different types of passive flights, namely: thermal soaring, linear soaring, slope soaring, gliding, and spiral gliding. Figure 2 illustrates all the flight types.

*Thermal soaring* is characterized by a circular flight in the course of which birds gain altitude in thermal columns using tight curves as close as possible to the centre (Pennycuick 1973, 2008; Videler 2005). The term *linear soaring* was introduced by Pennycuick (1972). It refers to an almost straight flight without circling when thermal currents are strong and abundant (Videler 2005). Although this term was introduced to describe long-distance flight, we have here applied the term to straight flight with a minimum length of 350 m to discriminate it from slope soaring. *Slope soaring* is a flight type often exhibited by Griffon vultures along their nesting or roosting cliffs. Generally, slope soaring takes place at a low altitude. Birds repeat this type of flight parallel to the cliff. This type of flight lasts until they detect a thermal or other air current (Barrios and Rodríguez 2004; Pennycuick 1972). It is performed in a shape that can be likened to a figure of eight. *Gliding* refers to flight with wings spread (or folded) in a downward or straight direction (Dhawan 1991; Pennycuick 1971, 2008). *Spiral gliding* is used to reduce altitude in an almost spiral-like pattern, and in slow downward motion towards the ground or to the nesting site. The term spiral gliding is not commonly used in the ornithology literature; it was borrowed from a study that focused on the flight behaviour of seeds dispersed by the wind (see: Minami and Azuma 2003).

### Flight Classification

To discriminate between linear flight patterns (i.e., linear soaring and gliding) and non-straight flight patterns (i.e., thermal soaring, slope soaring and spiral gliding), we calculated the radius of curvature parameter using a minimum of three successive GPS fixes. To further differentiate patterns within each flying type, we applied the laws of motion, as defined in physics, based on the following parameters: distance, altitude, speed and angle of direction.

For the space curve (like a non-straight flight pattern), the radius of curvature is the length of the curvature vector. To calculate the radius of curvature, we combined the flight distance and speed within non-straight flight patterns. In this regard, speed was smoothed with a running mean over three successive GPS fixes.The curvature *k* is defined as:

1 $$k = \frac{\Delta \phi }{\Delta s} = \frac{{\phi_{i + 1} - \phi_{i} }}{{s_{i + 1} - s_{i} }}$$

where *ϕ* denotes the tangential angle and s is the arc length. In three-dimensional space, the space curve *r*(*t*) for the tangent vector $\hat{T}$ is defined as:

2 $$\hat{T} \equiv \frac{{\frac{{\Delta \varvec{r}}}{\Delta t}}}{{\left| {\frac{{\Delta \varvec{r}}}{\Delta t}} \right|}} = \frac{{\frac{{\Delta \varvec{r}}}{\Delta t}}}{{\frac{\Delta s}{\Delta t}}} = \frac{{\Delta \varvec{r}}}{\Delta s}.$$

According to the Frenet–Serret formula, in differential geometry, keeping $\hat{T}$ as the tangent vector and $\hat{N}$. is the normal vector (Coxeter 1969) then we have:

3 $$\hat{\ddot{r}} = \hat{T}$$

4 $$\hat{\ddot{r}} = k\hat{N}.$$

When $\hat{\ddot{r}}$ changes constantly, it will show a circular flight (such as thermal soaring). However, if $\hat{\ddot{r}}$ fluctuates by showing increasing and decreasing magnitude, the flight can be considered to be slope soaring with its radius of curvature going up and down.

Another parameter that assisted in discriminating between non-straight flights was altitude, which constantly increases in thermal soaring and decreases in spiral gliding. However, it remains almost steady during the slope-soaring movement (*z* ~ 0).

**Fig. 3:**
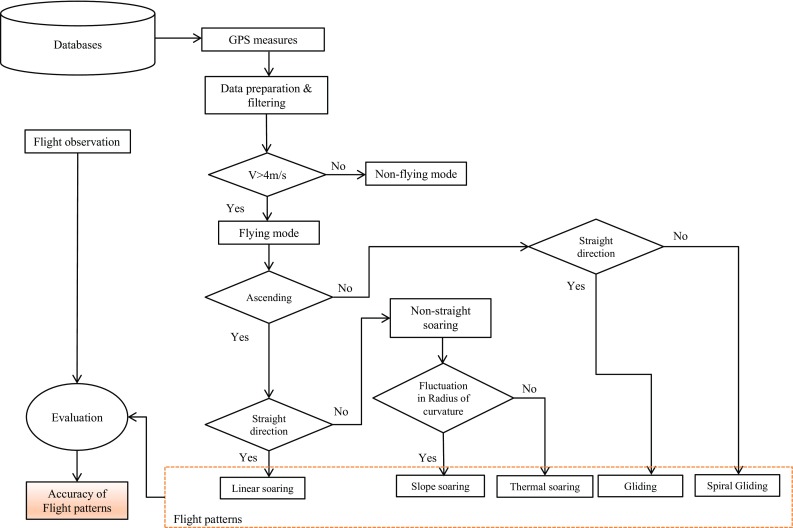
Study workflow of the Griffon vulture’s flight patterns and evaluation of the classification

Flights with a radius of curvature > 350 m were considered straight flights. To determine whether a flight pattern was soaring or gliding, regardless of whether it was straight or non-straight, the altitude of five successive GPS fixes (over a period of 15 s) were also considered. In this regard, soaring or gliding were characterized when the majority of the fixes (*n* ≥ 3) were either ascending or descending, respectively. Figure 3 shows the steps we took in building and evaluating our differentiation of Griffon vulture flight types.

### Evaluation of Flight Pattern Differentiation

An independent observation dataset was gathered during the fieldwork and used to evaluate predicted flight patterns. For this purpose, we collated and compared records based on the field observations and extracted 1146 s of flight synchronized with the final dataset. Considering each interval between two successive GPS points is 3 s, the length of recording consisted of 382 segments in total, matched with the dataset. It consisted of 54, 104, 109, 23 and 92 segments for linear soaring, gliding, thermal soaring, spiral gliding and slope soaring, respectively.

Validated results are presented in the form of a confusion matrix, (for example see: Kohavi and Provost 1998) giving the number of cases that were correctly classified as positive (i.e., predicted flight pattern), as well as the number correctly identified as negative (other flight patterns). The cases where a negative sample was misclassified as positive, and vice versa, are called false positive and false negative, respectively. The performance of the identified flight patterns was evaluated based on indicators, namely precision, true-positive rate, true-negative rate, the accuracy of each flight pattern, and the overall accuracy, as well as the kappa value (Weiss and Provost 2001) (see below for definitions).

Precision is defined as the proportion of the predicted cases that were correct. The true-positive rate indicates that the percentage of a flight pattern matches what is also observed from the data, while the true-negative rate expresses the proportion of other flight patterns that are correctly predicted as that class. The accuracy of each flight pattern is the proportion of predictions (positive or negative) that are correct. Overall accuracy is calculated by the total number of correct classifications divided by the total number of samples. Finally, the kappa value is used to measure the agreement between predicted and observed classes, while correcting for an agreement that might occur by chance (Stehman 1997; Viera and Garrett 2005). The confusion matrix (Table 1, left) shows the number of segments belonging to each flight pattern. For instance, in the first row, 41, 0, 7 and 6 are number of segments corresponding to each flight pattern classified as linear soaring, gliding, thermal soaring, spiral gliding and slope soaring, respectively. The numbers in diagonal line (in bold) are those segments that were correctly classified as positive.

**Table 1:** Summary statistics of confusion matrix for Griffon vulture’s flight patterns

Observed	Predicted flight pattern					Total observation	Classification’s performance indicators					
Flight pattern	Linear soaring	Gliding	Thermal soaring	Spiral gliding	Slope soaring		Precision (%)	True positive rate (%)	True negative rate (%)	Accuracy (%)	Kappa	Overall accuracy (%)
Linear soaring	**41**	0	7	0	6	54	83.67	75.92	97.56	92.73		
Gliding	0	**81**	0	12	11	104	88.04	77.88	90.04	88.74		
Thermal soaring	5	0	**89**	0	15	109	75.42	81.60	89.37	85.50		
Spiral gliding	3	0	0	**16**	4	23	34.78	69.56	91.64	87.86		
Slope soaring	0	11	22	18	**41**	92	53.24	44.56	87.58	75.41		
Total predicated	49	92	118	46	77	382					0.61	70.15

The columns and rows (left side) show the predicted and observed flight patterns, respectively. Numbers are representatives of segment. The numbers in bold are corresponding segments of each flight pattern which were correctly classified as positive. Summary statistics of the classification’s performance (right side) for all flight classes, overall performance of the classification

## Results

The evaluation method indicated a substantial agreement between the predicted and observed Griffon vulture’s flight types (Table 1, right). The estimated kappa value (0.61 ± 0.06) is intended to illustrate the agreement between two groups of predicted and actual flights. The overall classification accuracy was 70%. Of all flight patterns, gliding had the highest precision (88%), while linear and thermal soaring had a precision of 83 and 75%, respectively. The lowest values of precision were present for spiral gliding (34%) and slope soaring (53%).

**Fig. 4:**
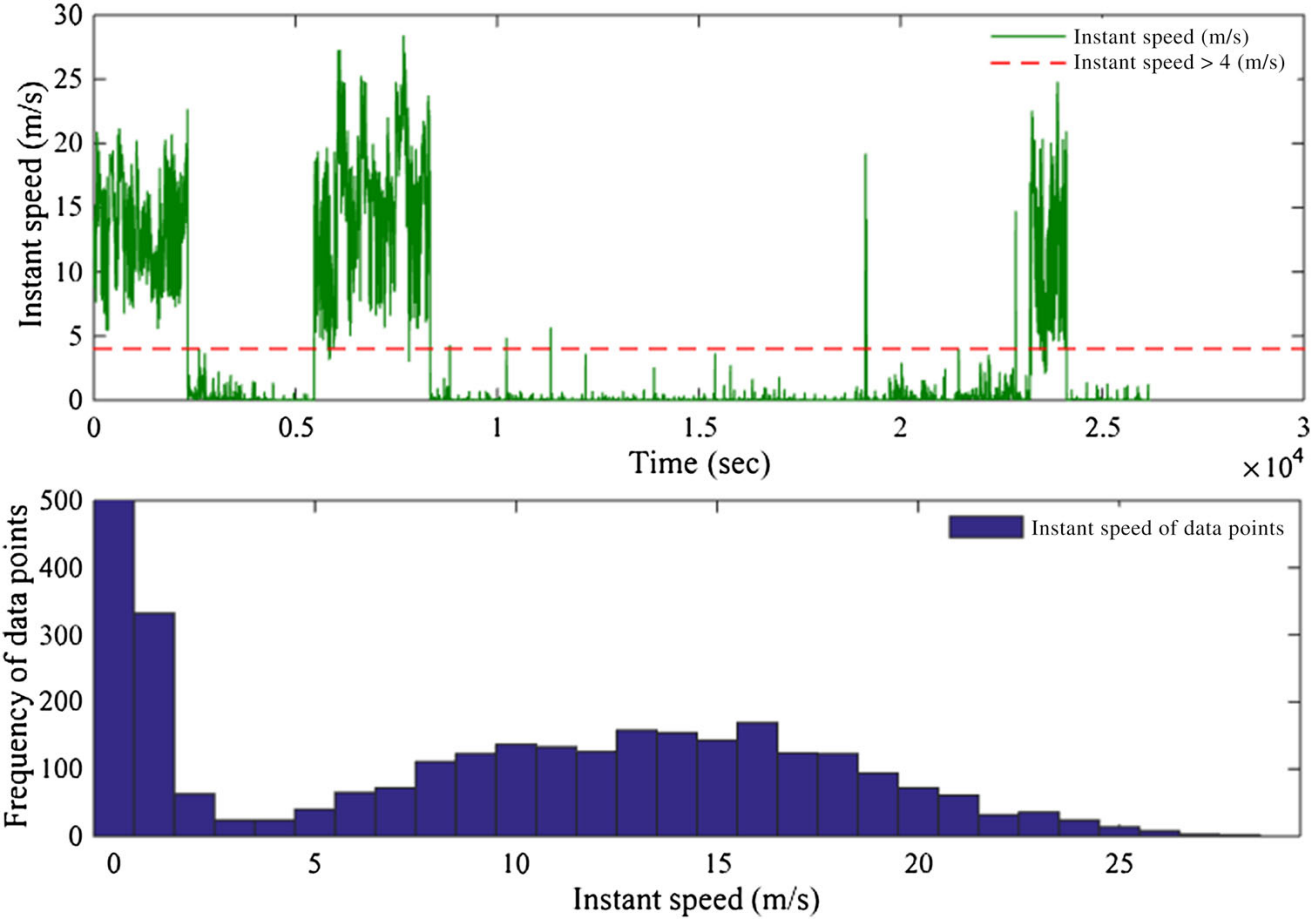
Variation and frequency of instant speed in the dataset: **a** instantaneous speed > 4 m/s (red dashed line) is the main proxy to identify flying mode, and **b** frequency of flying and static modes in the dataset

The flying and stationary modes were clearly distinguished. The variation of instantaneous speed > 4 m/s, as a main proxy of the flying mode, is demonstrated in Fig. 4. This figure also shows that the stationary mode is more frequent than flying mode in the period of our study.

Although thermal and linear soaring, as well as gliding, were classified correctly to a high degree of the estimated precision, some misclassifications of flights also occurred. Linear soaring was mostly misclassified as thermal soaring. Gliding was also misidentified as slope soaring, while spiral gliding was misclassified as either gliding or slope soaring. Finally, slope soaring was mixed up with thermal soaring, gliding, and linear soaring. Slope soaring and spiral gliding had the lowest values of the true-positive rate. The highest true-positive rate (81%) was achieved for thermal soaring at 81% and was slightly better than that for linear soaring or gliding. The true-negative rates were excellent for all flight patterns. The lowest and highest values of true-negative rate were achieved for slope soaring (85%) and linear soaring (97%), respectively. The predicted accuracy measures, and the proportion of positives or negatives were excellent for all flight patterns.

Linear soaring (92%) and slope soaring (75%) were the most and least accurate flight types, respectively.

**Fig. 5:**
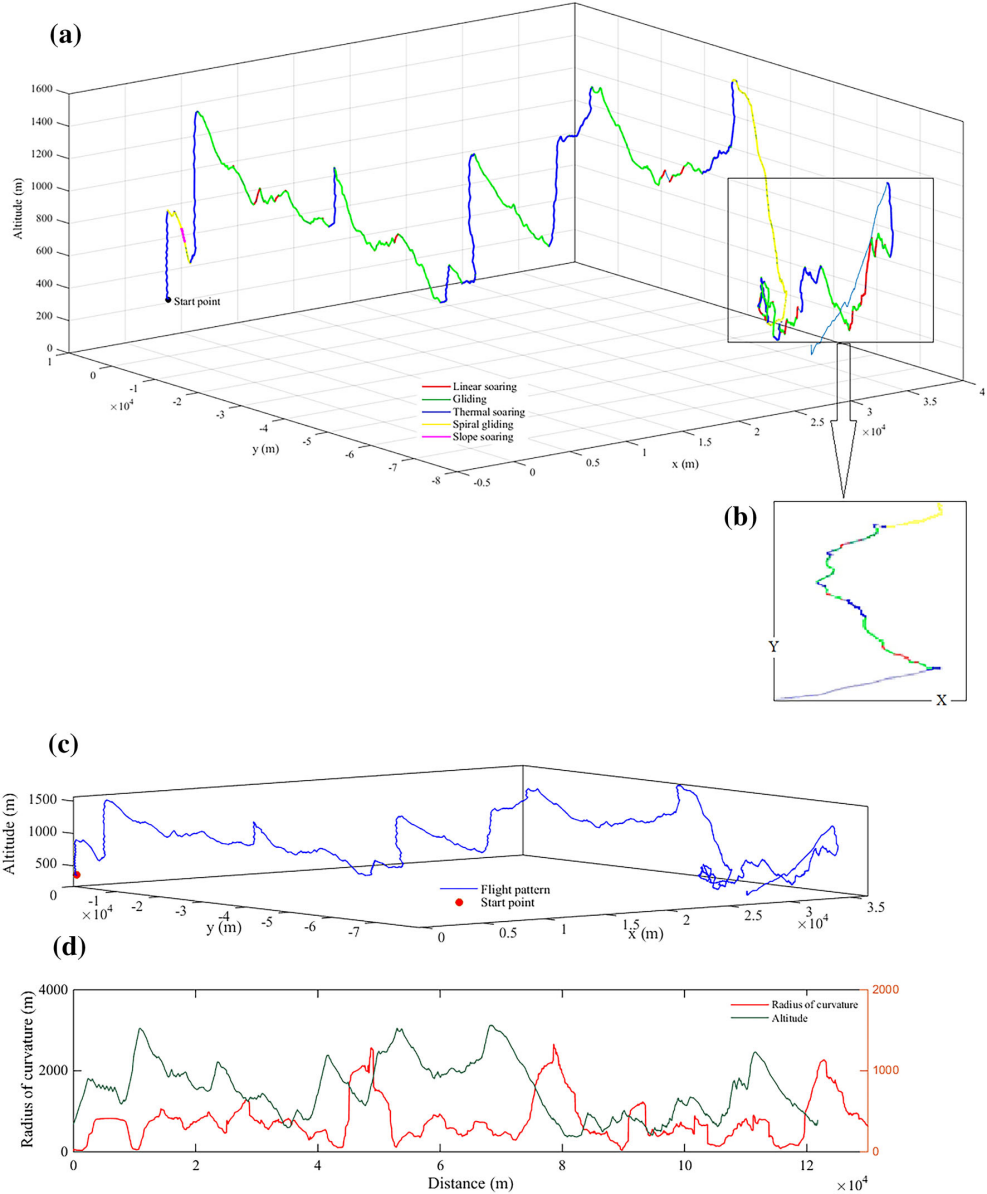
**a** Scheme of the Griffon vulture’s flight patterns in three dimensions, and **b** in two dimensions segregated using the concept of motion in physics. **c** Scheme of the Griffon vulture’s flight patterns in three dimensions, and **d** its relative radius of curvature (red line) and altitude (green line) during the flight

Examples of the different flight types in three dimensions are visualized in Fig. 5a, b, demonstrating variation of flight behaviour in different altitude and with the use of thermal soaring the bird reached up to 1400 m above sea level. Additionally, Fig. 5c, d shows a scheme of radius changes as the bird flew along the curve.

## Discussion

Our study differentiated five passive flight types of the Griffon vulture including linear soaring, thermal soaring, slope soaring, gliding and spiral gliding. To our knowledge, this is the first reported differentiation of a raptor’s flight patterns using tracking data. Our results show differences between flight patterns in terms of accuracy, precision, true-positive rate, and true-negative rate. Each class shows over 75% performance in accuracy. Due to the unbalanced structure (the ratio of positive and negative cases, the predicted flight and other flight pattern) in most of the observed data, other measures of the classification’s performance, such as precision and true-positive rate, are more informative (Kubat et al. 1998; Martiskainen et al. 2009).

The classification precision was high for linear and thermal soaring as well as for gliding. The lower precision values seen for spiral gliding and slope soaring indicate that the classification method can be problematic in predicting positive cases (predicted flight pattern) correctly. Most cases of confusion involved slope soaring. This may be because it could be the most complex flight pattern, or because it closely resembles other patterns. Part of the difficulty could lie in the sampling rate of the flight type, which might have been too low to discriminate slope soaring well enough. This is in fact supported by the Nyquist Theorem (also known as the sampling theorem), according to which the minimum sampling rate must be twice the highest frequency contained in the flight pattern (Grenander 1959).

Our results reveal that the highest percentage of misclassification is seen for spiral gliding, due to the inadequate number of samples (Bohrer et al. 2012; Kubat et al. 1998; Mellone et al. 2015) in our current dataset. Since only two fieldworkers were assigned to collect the observational dataset in a limited time, there may also have been some human error during sightings or recording the bird’s flight behaviour and this might have affected the dataset.

The true-positive rate was high in the three flight patterns of linear soaring, thermal soaring and gliding. This implies that fewer negative cases (predicted other flight pattern) were falsely classified in those flight patterns; in other words, the true-positive rate shows these three flight types were more often correctly identified than slope soaring and spiral gliding. The excellent values (85% and higher) of the true-negative rate in all the flight types also shows that the negative cases were correctly classified for those flight patterns. The value of kappa (0.61 ± 0.06) shows a substantial classification agreement, which could be interpreted as demonstrating the method’s success.

For the above flight types, data with finer temporal resolution (e.g., a 1 s interval) of GPS fixes might be useful for making a more precise and accurate classification. In this experiment, although we set the measurement interval of the GPS tracker to 3 s, the retrieved dataset consisted of unequal intervals. By filtering out the coarse temporal resolution, some gaps in the dataset decreased the consistency of the data. Due to the varying success in classifying the flight types, it might be worthwhile to include various parameters (e.g., time window) in the classification process. More specifically, including other parameters (e.g., aspect ratio or wing loading) would entail considering the traits of each flight pattern. Another point that could improve classification performance is the further optimization of different flight characteristics (e.g., horizontal vs. vertical speed).

Since we can assume birds’ flight types are affected by the environmental conditions (Shamoun-Baranes et al. 2004) when gathering data in different seasons, there may be an opportunity to observe and digitally capture more flight types, particularly those that were seen less often during the period of this study. We speculate that the Griffon vulture spent more time in the non-flight mode because we performed the study during the breeding season; the birds would have been in parental mode and more vigilant than at other times of the year to protect their chicks from bad weather and predators (Xirouchakis and Mylonas 2009).

## Conclusion

This study investigated the flight types of the Griffon vulture using high-resolution GPS data and we provide evidence that such data contains sufficient information to recognize Griffon vulture’s flight types. In movement ecology research, our study makes a useful contribution by providing a new baseline technique using GPS sensor data to classify a bird’s flight type as a part of its motion capacity. However, more studies are needed to refine the properties employed in this classification method, including the testing of other types of sensory data (e.g., accelerometer data) or the use of different analytical parameters. Collision risk of the Griffon vulture was mediated by flight behaviour and it is suggested that a detailed research on flight behaviour is needed at precise location where the turbines are installed (Barrios and Rodríguez 2004), so our study is one step forward to solve the collision dilemma.

## Appendix

See Table 2.

**Table 2:** Details of studies pertaining to movement and foraging of Griffon vulture

Subject	Method	Major findings	References
Flight and foraging behaviour	Electrocardiogram, GPS and accelerometers	Heart rate increased three-fold during take-off and landing compared to baseline level. 10 min after initial flapping phase, home range in soaring and gliding dropped to the baseline level that was lower than theoretically possible	Duriez et al. (2014)
	GPS tracking	Change in size of home range in different seasons and also amongst individuals. Vultures prefer a feeding station compared to the rest of the habitat with unpredictable food resources	Monsarrat et al. (2013)
	GPS and accelerometers	Despite high variability in food deprivation periods, flight speed, straightness of flight and the proportion of active flights do not vary in relation to food deprivation	Spiegel et al. (2013)
	GPS and tri-axial accelerometer	Classifying behavioural modes using machine learning classifiers with 80–90% accuracy	(Nathan et al. 2012)
	GPS satellite telemetry	Traditional stock-raising areas are the Griffon vultures’ main range. Overall foraging range is 1719 km^2^ as minimum convex polygon, with 4078 and 489 km^2^ as 95 and 50% kernel contours, respectively	García-Ripollés et al. (2011)
	GPS and tri-axial accelerometer	Hungry individuals (fasted > 4 days) spent more time flying, travelled longer distances, and their paths were less straight than well-fed ones	(Harel et al. 2010)
	Radio telemetry and direct observation	Griffon vultures spend 7.6 h/day on food searching, mean distance from colony to feeding area is 8.4 km, mean foraging radius is 15 km, foraging ranges, based on direct observations are 206–851 and 195–527 km^2^ using the adaptive kernel method. The range based on radio tracking is 390–1300 km^2^	Xirouchakis and Mylonas (2009)
	Tri-axial accelerometer	Griffon vultures use legs before taking off and after landing. Mean overall dynamic body acceleration for flying up and down a hill were 1.396 ± 0.114 and 0.889 ± 0.123, respectively	Halsey et al. (2009)
	Direct observation	Finding food directly or relying on following other birds, food searching is concentrated on large ungulate herds, gaining altitude with lower density of ungulates in a herd	Houston (1974)
Migration flying characteristics	Direct observation	Higher rate of flapping when crossing water than land, flapping rate and attempts to cross water are influenced by time and weather conditions, passage over a water body is limited by Griffon vulture’s over-water flapping-flight abilities	Bildstein et al. (2009)
	Satellite tracking	A Griffon vulture changed migration direction from south to north and its longest flight distance in a day was 80 km	Berthold et al. (1991)
